# Drug Prices After Patent Expirations in High-Income Countries and Implications for Cost-Effectiveness Analyses

**DOI:** 10.1001/jamahealthforum.2024.2530

**Published:** 2024-08-16

**Authors:** Miquel Serra-Burriel, Nicolau Martin-Bassols, Gellért Perényi, Kerstin N. Vokinger

**Affiliations:** 1 Faculty of Law and Faculty of Medicine, University of Zurich, Zurich, Switzerland; 2 Epidemiology, Biostatistics, and Prevention Institute, University of Zurich, Zurich, Switzerland; 3 Department of Economics, University of Bologna, Bologna, Italy; 4 Department of Mathematics, Ecole Polytechnique Fédérale de Lausanne, Lausanne, Switzerland

## Abstract

**Importance:**

Understanding how patent expirations affect drug prices is crucial because price changes directly inform accurate cost-effectiveness assessments. This study investigates the association between patent expirations and drug prices in 8 high-income countries and evaluates how the changes affect cost-effectiveness assessments.

**Objective:**

To analyze how the expiration of drug patents is associated with drug price changes and to assess the implications of these price changes for cost-effectiveness evaluations.

**Design, Setting, and Participants:**

This cohort study performed an event study design using data from 8 high-income countries to assess the association between patent expiration and drug prices, and created a simulation model to understand the implications for cost-effectiveness analyses. The simulation cost-effectiveness model analyzed the implications of including or ignoring postpatent price dynamics.

**Exposure:**

Drug patent expiration.

**Main Outcomes and Measures:**

Change in drug prices and differences in incremental cost-effectiveness ratios when considering vs ignoring postpatent price dynamics.

**Results:**

The sample comprised 505 drugs undergoing patent expiration in Australia, Canada, France, Germany, Japan, Switzerland, UK, and US. Price decreases were statistically significant over the 8 years after patent expiration, with the fastest price declines observed in the US: 32% (95% CI, 24%-39%) in year 1 after patent expiration and 82% (95% CI, 71%-89%) in the 8 years after patent expiration. Estimates for other nations ranged from a decrease of 64% in Australia to 18% in Switzerland in the 8 years after expiration. The cost-effectiveness simulation model indicated that not accounting for generic entry into the market may produce biased incremental cost-effectiveness ratios of 40% to −40%, depending on the scenario.

**Conclusions and Relevance:**

The findings of this cohort study demonstrate that drug prices were reduced substantially after patent expirations in high-income countries. Therefore, incorporating information on patent status and pricing dynamics in cost-effectiveness assessment analyses is necessary for producing accurate economic evaluations of new drugs.

## Key Points

**Question:**

How do drug prices change after patent expirations, and how do these price changes affect cost-effectiveness analyses?

**Findings:**

This cohort study using data from over 500 drugs losing patent protection between 2011 and 2020 in 8 high-resource economies (Australia, Canada, France, Germany, Japan, Switzerland, UK, and US) found sizable price decreases associated with patent loss, which varied substantially across countries. Incorporating these estimates into simulated cost-effectiveness models showed that depending on the scenario (generic or patented comparator), large differences in incremental cost-effectiveness ratios happen when omitting the postpatent price dynamics.

**Meaning:**

These findings quantify the decrease of drug prices after patent expiration and indicate that ignoring these estimates may produce biased cost-effectiveness estimates.

## Teaser

This economic evaluation assesses drug price changes after patent expirations in 8 high-income countries and what the implications are for cost-effectiveness analyses.

## Introduction

The loss of patent protection for originator small molecule drugs and biologics (hereafter, drugs) is a crucial event for manufacturers, patients, and health care systems. After a period of profit internalization by the innovator, society benefits from the innovation at lower prices due to the market entry of generic versions and the subsequent price competition.^1^ However, the level of competition after market entry of generic and/or biosimilar drugs (hereafter, generics) varies strongly across markets and nations.^2,3^

Cost-effectiveness analyses (CEAs) of new drugs are conducted in many countries as a basis for price negotiations and reimbursement decisions, aiming to improve the efficiency of health care systems.^4^ CEAs are based on comparative analyses; that is, analysts compare the costs and health effects of the new originator drug with those of the standard of care, which may be a drug with the same indication. The price of therapeutic alternatives tends to be the most important contributing factor when determining the cost-effectiveness profiles of drugs. Almost all new originator drugs are under patent protection when they become available, and expiration follows after market entry.^5^ A previous study^6^ showed that generics enter the market an average of 14 years after approval of the originator drug. Most CEAs assume fixed, nondynamic prices for the compared drugs. If the comparator drug is a generic, and the CEA does not account for the originator reduction in its price, the analysis can overestimate the originator’s cost-effectiveness ratio (ie, produce an unrealistically unfavorable ratio for the originator). In contrast, if the comparator drug is also under patent protection and its price is assumed to remain constant, the CEA may underestimate the cost-effectiveness ratio (ie, an unrealistically favorable ratio for the originator).

Prior studies have criticized the assumption that prices will remain constant^7^ because this approach does not adequately reflect reality, and there have been debates regarding the incorporation of drug price dynamics in CEAs.^8,9^ Some authors argue that it is crucial to consider future price dynamics into CEAs.^10^ By not incorporating price changes, researchers fail to capture the true economic value and cost-effectiveness of drugs over time. This may produce biased estimates that do not reflect the actual costs incurred by health care systems. Moreover, when CEAs are used to decide coverage of treatments, not accounting for price changes affects decisions on resource allocation. In contrast, other authors^11^ highlight that cost-effectiveness estimates are contingent on the specific context of the patient cohort that will receive the treatment and the associated economic context that the decision-maker is facing. These authors argue that future price dynamics are better addressed with further evaluations as prices changes. This argument has been criticized in the context of chronic disease therapies with lifelong administration patterns.^12^

Nonetheless, there is a lack of estimates of pricing dynamics before and after the patent expiration of the new originator drug or comparative drug, and unclarity of their general association with cost-effectiveness estimates.^13,14^ A recent review^15^ assessed to what degree there is clear guidance on this issue, concluding that the omission of assumptions regarding generic entry misguides long-run CEAs and highlights the need for further work in the area.

This economic evaluation sought to analyze how the expiration of drug patents is associated with drug price changes, and to assess the implications of these price changes for cost-effectiveness analyses. Price developments of new originator drugs before and after patent expiration were compared with drugs that did not experience patent expiration simultaneously in 8 large and high-income economies. Furthermore, through simulation studies, we assessed the effects of incorporating and ignoring the postpatent price dynamics of originator and comparator drugs into cost-effectiveness models.

## Methods

This study was exempt from institutional review board approval and the need for informed consent because it used proprietary nonidentifiable data that did not constitute research with human participants (in accordance with US Department of Health and Human Services 45 CFR § 46.102). We followed the Strengthening the Reporting of Observational Studies in Epidemiology (STROBE) reporting guideline and the Reporting Guidelines for Health Care Simulation Research (SBR).

### Study Design

The study was designed as 2 distinct parts. First, we conducted an empirical study of price developments for drugs before and after patent expiration compared with patented drugs at the same time in 8 countries (Australia, Canada, France, Germany, Japan, Switzerland, UK, and US). Second, we constructed a cost-effectiveness model to simulate how patent expiration of originator and comparator drugs impacted pricing estimates of new drugs.

### Data Collection

We used drug pricing data from the IQVIA (formerly QuintilesIMS) MIDAS database for the time period from 2011 to 2020 for 8 economically comparable countries—Australia, Canada, France, Germany, Japan, Switzerland, UK, and US. The dataset contained quarterly information on list prices, units, companies, molecules, doses, and patent status for the included drugs. For the included countries, both retail and in-hospital sales data were available. Our unit of observation was at the molecule level and the outcome was the unit-weighted average price for a given quarter. eTable 1 in Supplement 1 presents the descriptive statistics of the analytical sample by included country, and eTable 2 in Supplement 1 displays the number of available observations with respect to the timing of the patent expiration alongside the patent status.

In our analytical sample, we included all drugs under patent protection at the start of the observation window. We classified drugs that lost their patent at any time point between 2011 and 2020 as the treatment group, and those drugs that did not lose their patent in this time period in the control group. The data contained list prices adjusted for national inflation; we were not able to account for confidential rebates.^16^

### Statistical Analyses

To assess the association between patent expiration and drug prices, we used the timing of patent expiration within each of the 8 countries between 2011 and 2020. We used the quasi-experimental variation created by the staggered loss of patents. We used a difference-in-differences strategy, which under a certain set of assumptions allowed us to approximate the unbiased association between patent expiration and drug prices dynamics. We estimated 1 model per country, independently.

The main assumption for our empirical strategy was that if drugs did not experience patent loss, their prices would have evolved in parallel to those under patent protection. Under this assumption, our model would adjust for constant price differences across drugs and common national pricing trends. To provide empirical support for this assumption, we estimated a dynamic version of an event study to examine the existence of pretrends with the Callaway and Sant’Anna difference-in-differences estimator.^17^ Another reason to use the estimator instead of a standard 2-way fixed-effects model was that if the treatment effects were heterogeneous across time, the standard difference-in-differences estimator would be inconsistent. The target estimate was the average treatment effect on the treated group (ATT), ie, the drug price changes associated with losing patent for those drugs whose patent expired. We clustered the standard errors of the coefficients of interest at the molecule level.

To assess the degree of potential bias due to measurement error (difference between list prices and unobserved net prices), we performed a sensitivity analysis controlling for time-varying volume of sales. Statistical tests were 2-tailed and *P* < .05 was considered statistically significant. Data analyses were performed from March 3, 2023, to June 20, 2023, using R, version 4.2.2 (R Core Team).

### Theoretical Cost-Effectiveness Modeling and Simulation Models

To assess the influence of price dynamics after patent expiration on cost-effectiveness estimates, we created a simulation model focusing on 2 scenarios. In the first scenario, the originator drug was subject to patent protection for a time period before patent expiration, while the comparator drug was a generic. This first scenario, which assumed a constant price for the comparator, could also be interpreted as a drug vs nondrug comparative analysis. In the second scenario, both drugs—the originator and the comparator drug—were under patent protection, with the latter losing patent protection before the originator drug. For both scenarios, the outcome of interest was the difference in incremental cost-effectiveness ratios (ICERs) between the case where prices are allowed to change over time vs the case where prices are assumed to be constant.

The motivation for our modeling approach was due to most economic evaluations of drugs occurring when a new originator drug enters the market. In most cases, the new drug is under patent protection at market entry but will lose its patent within the modeling time horizon. Our model included incremental costs and survival effects discounted at a constant rate over the time horizon:
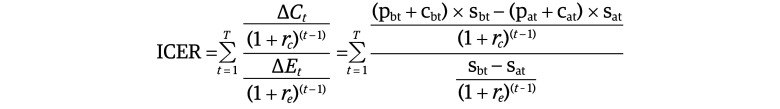
in which incremental effectiveness ΔE*_t_* is expressed as the difference in survival rates s_bt_−s_at_ between intervention *b* (originator) and *a* (comparator), at time point *t.* Incremental costs Δ*C_t_* are defined as the difference in drug price p_t_ plus nondrug costs c_t_ (eg, medical labor costs) times the proportion of patients that survived S_t_ (assuming that patients receive therapy while alive) between interventions *b* and *a.* Both incremental costs and survival effects are discounted at *r_c_* and *r_e_*, respectively. Derivations and special cases of the model are presented in the supplementary cost-effectiveness model summary. Note that this model can be interpreted as a drug vs drug comparative effectiveness model, but also as a drug vs nondrug model when drug prices for *a* are set to 0.

Then we incorporated the pricing estimates from the empirical assessment at 2, 8, and 14 years after target exposure introduction times. We presented 3 effectiveness scenarios with different survival effects hazard ratios (HRs, 0.44, 0.58, and 0.89)^18,19^ to represent high-, mid-, and low- effectiveness levels, respectively. Then we assessed the extent of the influence that the introduction of pricing dynamics has depending on the above-mentioned scenarios. Additionally, we created an online open-access web tool (Incremental Cost-Effectiveness Analysis with Post-Patent Pricing tool) that can be used to explore the influence of parameters and scenarios.

## Results

### Drug Prices

The analysis included a total of 505 unique originator drugs: 497 in the US, 359 in the UK, 323 in Switzerland, 355 in Japan, 378 in Germany, 320 in France, 361 in Canada, and 338 in Australia. Across all countries, most of the drugs were antineoplastic or immunomodulating agents (eTable 1 in Supplement 1).

Of all studied countries, the US had the highest mean and median drug prices throughout the study period. Mean list prices in the US were between 3.5 (Japan) and 1.6 (Germany) times higher for new originator drugs under patent protection, and 4.1 (Australia) and 1.7 (Switzerland) times higher for drugs without patent protection. The pattern was similar for median prices.

Regarding price developments of patent-protected drugs, the US experienced the highest mean price increase between 2011 and 2020—119% growth; whereas in the other countries, the increase ranged from 10% to 30%. eTable 2 in Supplement 1 provides the summary statistics of prices, and eTable 3 in Supplement 1 shows the distribution of observations with and without patent expiration at different time points.

### Event Study

**Figure 1.**
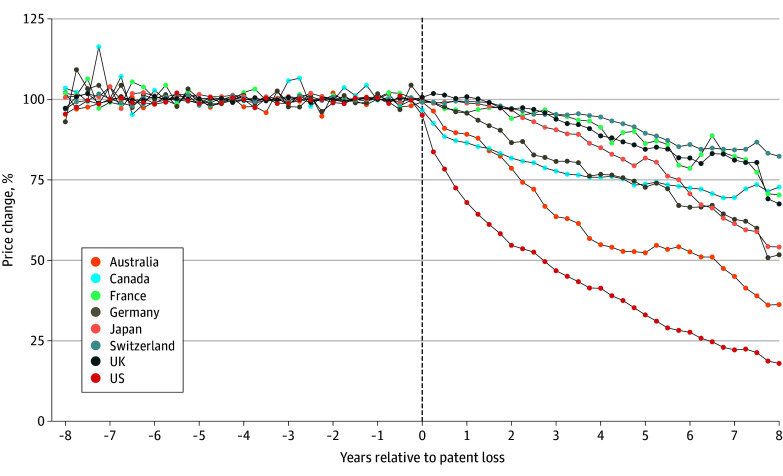
Difference-in-Differences Dynamics Estimates, by Country Confidence intervals are not displayed for comparison purposes and are available in eFigure 1 in Supplement 1.

After patent expiration, drug prices decreased in all countries. The dynamic ATT estimates, by country, are depicted in Figure 1. Our preintervention estimates also provide strong support for parallel trends in all countries, up to 8 years prepatent loss (eFigure 1 in Supplement 1).

We observed stark differences across countries regarding effect sizes. The US and Australia presented the steepest declines in prices, reaching 82% and 64% during the 8 years after patent expiration, respectively. Japan and Germany followed with an approximately 50% price decreases. Canada, France, the UK, and Switzerland presented the smallest price decreases with 27%, 30%, 32%, and 18%, respectively, 8 years after generic entry. The Table presents the numeric estimates and confidence intervals for each country, and eFigure 1 in Supplement 1 presents all estimates of the countries with their respective confidence intervals. Additionally, no major differences between the preferred specification estimates and the time-varying volume control were found overall for the included countries (eFigures 2-9 in Supplement 1), with the exception of relatively more conservative estimates for Australia and Canada.

**Table.** Drug Price Change Estimates During Years Before and After Patent Expiration

Years	Country, Exp-β (95% CI)							
	Australia	Canada	France	Germany	Japan	Switzerland	UK	US
−8	1.02 (0.96-1.08)	1.03 (0.93-1.15)	1.02 (0.97-1.07)	0.93 (0.75-1.15)	1.01 (0.99-1.03)	0.97 (0.88-1.08)	0.97 (0.88-1.08)	0.95 (0.89-1.02)
−7	1.04 (0.95-1.14)	1.00 (0.98-1.02)	0.99 (0.95-1.04)	0.99 (0.94-1.05)	1.04 (0.93-1.16)	1.00 (0.98-1.03)	1.00 (0.97-1.02)	1.00 (0.96-1.04)
−6	0.99 (0.95-1.03)	1.03 (0.96-1.1)	1.00 (0.98-1.03)	1.00 (0.95-1.05)	1.01 (0.99-1.04)	(0.99 (0.97-1.01)	1.01 (0.99-1.03)	(0.99 (0.96-1.01)
−5	1.00 (0.97-1.03)	0.99 (0.96-1.03)	0.99 (0.96-1.02)	1.00 (0.97-1.04)	1.02 (0.99-1.04)	0.98 (0.95-1.01)	1.00 (0.97-1.03)	0.99 (0.97-1.01)
−4	0.98 (0.94-1.02)	1.00 0.98-1.01)	1.02 (0.99-1.05)	1.01 (0.97-1.05)	1.01 (0.98-1.04)	1.00 (0.99-1.01)	1.00 (0.99-1.01)	1.00 (0.98-1.02)
−3	0.99 (0.96-1.02)	1.06 (0.98-1.14)	0.98 (0.96-1.00)	0.98 (0.92-1.04)	1.01 (0.98-1.04)	0.99 (0.98-1.01)	1.01 (0.99-1.02)	0.99 (0.97-1.01)
−2	1.02 (0.99-1.05)	1.00 (0.98-1.01)	1.01 (0.98-1.05)	0.99 (0.95-1.03)	1.00 (0.98-1.02)	1.01 1.00-1.02)	1.00 (0.99-1.01)	0.99 (0.97-1.01)
−1	1.00 (0.98-1.03)	1.00 (0.98-1.01)	1.00 (0.98-1.03)	1.00 (0.96-1.05)	1.00 (0.98-1.02)	1.00 (0.99-1.01)	1.00 (0.99-1.01)	1.00 (0.99-1.02)
0	0.99 (0.97-1.02)	0.97 (0.94-0.99)	1.00 (0.98-1.02)	1.00 (0.98-1.02)	1.00 (0.99-1.01)	0.99 (0.98-1.00)	1.01 (1.0-1.02)	0.95 (0.93-0.97)
1	0.89 (0.84-0.95)	0.87 (0.81-0.92)	0.96 (0.91-1.02)	0.96 (0.89-1.02)	0.99 (0.92-1.06)	0.99 (0.97-1.02)	1.01 0.98-1.04)	0.68 (0.61-0.76)
2	0.79 (0.72-0.86)	0.82 (0.75-0.89)	0.94 (0.86-1.03)	0.87 (0.78-0.96)	0.97 (0.87-1.08)	0.97 (0.93-1.01)	0.97 (0.93-1.01)	0.55 (0.47-0.64)
3	0.64 (0.55-0.73)	0.78 (0.70-0.86)	0.95 (0.85-1.06)	0.81 (0.70-0.94)	0.91 (0.80-1.03)	0.95 (0.91-1.00)	0.94 (0.89-0.99)	0.47 (0.38-0.57)
4	0.55 (0.45-0.67)	0.76 (0.66-0.87)	0.91 (0.78-1.06)	0.77 (0.64-0.92)	0.85 (0.73-0.99)	0.95 (0.88-1.01)	0.89 (0.83-0.95)	0.41 (0.33-0.52)
5	0.52 (0.41-0.67)	0.74 (0.64-0.85)	0.86 (0.72-1.03)	0.73 (0.58-0.92)	0.82 (0.68-0.98)	0.89 (0.82-0.97)	0.85 (0.78-0.92)	0.33 (0.25-0.44)
6	0.53 (0.41-0.67)	0.72 (0.62-0.84)	0.79 (0.63-0.98)	0.66 (0.52-0.86)	0.71 (0.57-0.87)	0.86 (0.77-0.96)	0.82 (0.73-0.91)	0.28 (0.20-0.38)
7	0.45 (0.34-0.60)	0.69 (0.59-0.83	0.82 (0.65-1.04)	0.63 (0.46-0.85)	0.61 (0.46-0.81)	0.84 (0.75-0.94)	0.81 (0.73-0.91)	0.22 (0.15-0.34)
8	0.36 (0.24-0.54)	0.73 (0.59-0.89)	0.70 (0.54-0.91)	0.52 (0.35-0.76)	0.54 (0.37-0.78)	0.82 (0.7-0.96)	0.68 (0.58-0.79)	0.18 (0.11-0.29)

Abbreviation: Exp-β, estimated coefficient

The control group was never treated, 0 anticipation periods, doubly robust estimation, Callaway-Sant’Anna staggered difference-in-differences method.

### Estimated Cost-Effectiveness Effects

**Figure 2.**
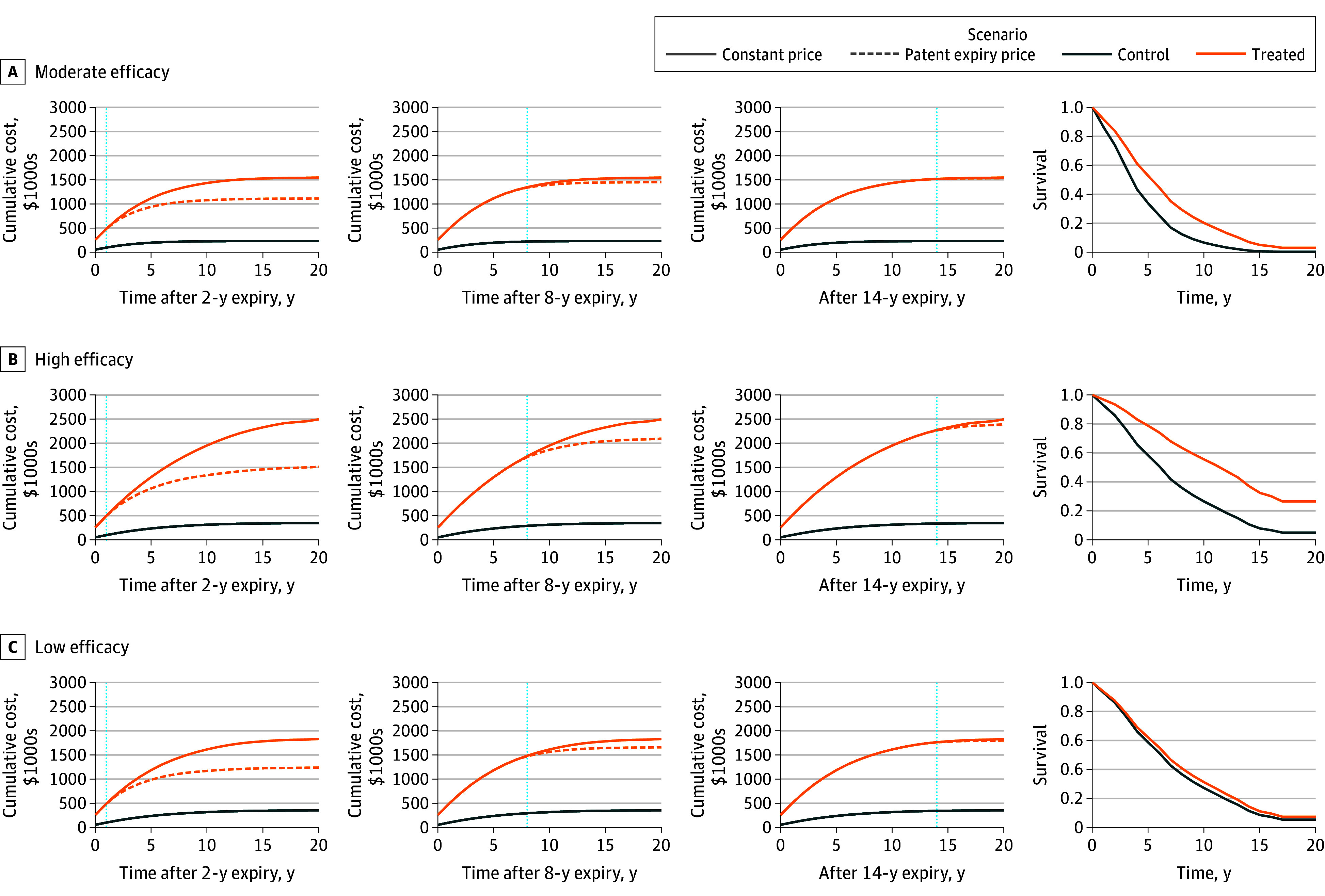
Cost-Effectiveness Simulation Scenarios With and Without Postpatent Expiry Prices for the New Originator Drug Parameters for the simulation: price of annual new treatment, $200 000; nontreatment annual cost, $50 000; and price of comparator, $1000. Scenarios A, B, and C present different survival (effectiveness profiles). Hazard ratio for scenario A was 0.58; for B, 0.44; and C, 0.89. Different columns present different times of patent expiry with relation to the investment assessment timing. The first column presents a scenario where the patented drug loses patent 2 years after introduction. The second column presents a scenario where the patent is lost 8 years after, and the third 14 years afterwards. The last column represents the survival profile of each treatment arm compared. For effectiveness, in scenario A, assuming constant prices vs dynamic ones produces an underestimation in the incremental cost-effectiveness ratios (ICERs) of 33% for the first case, 8% for the second case, and 1% for the third case; in scenario B, assuming constant prices vs dynamic ones produced an underestimation in the ICERs of 45% for the first case, 19% for the second case, and 5% for the third case; and in scenario C, assuming constant prices vs dynamic ones produces an overestimation in the ICERs of 40% for the first case, 12% for the second case, and 2% for the third case.

**Figure 3.**
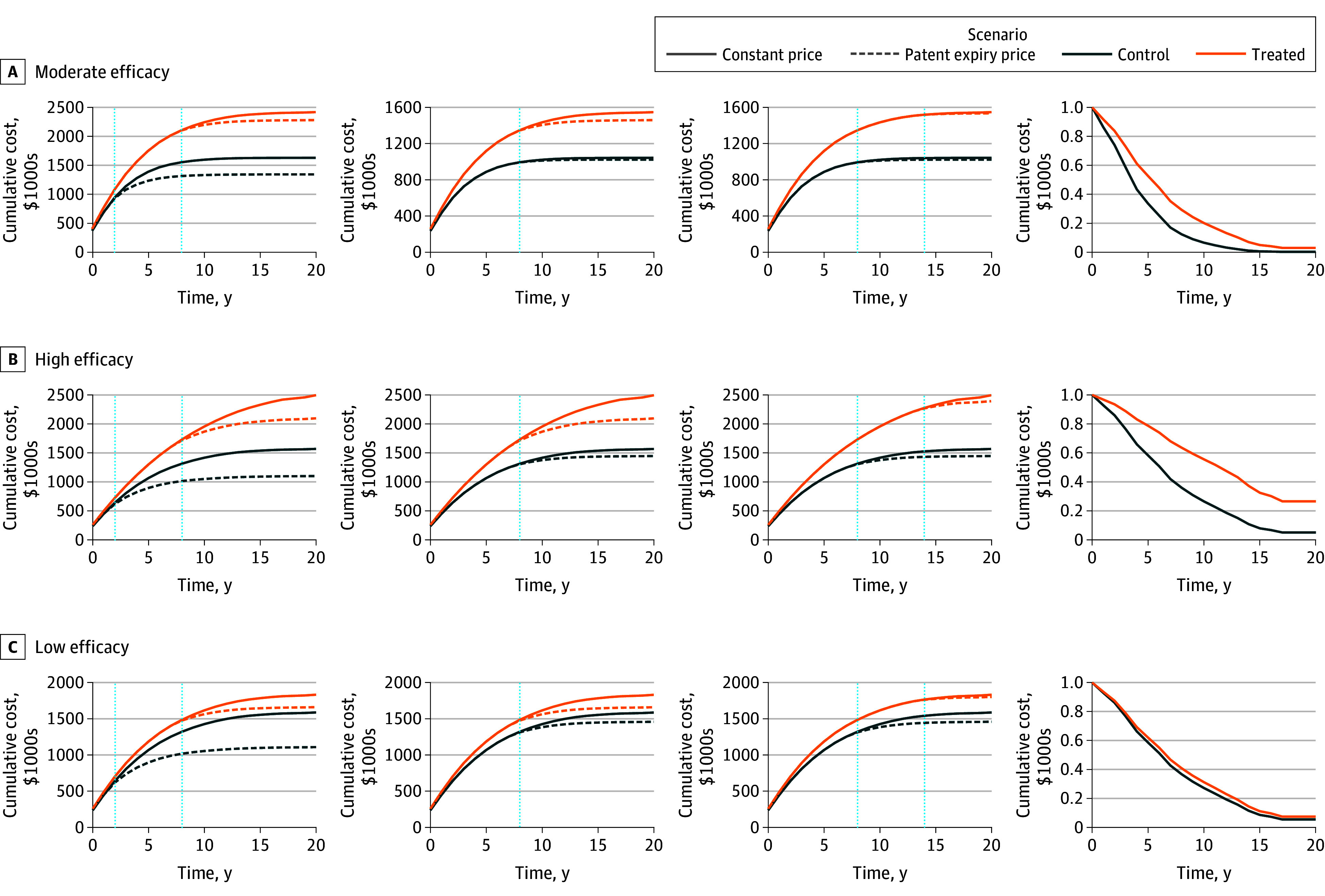
Cost-Effectiveness Simulation Scenarios With and Without Postpatent Expiry Prices for the New Originator Drugs and Their Control Arms Parameters for the simulation: price for annual new treatment, $200 000; nontreatment annual costs, $50 000; and price of comparator, $180 000. Scenarios A, B, and C present different survival (effectiveness profiles). Hazard ratio for scenario A was 0.58; for B, 0.44; for C, 0.89. Different columns present different times of patent expiry with relation to the investment assessment timing. The first column presents a scenario where the comparator is set to lose patent protection 2 years after the introduction of the new drug, while the new drug patent expires 8 years after introduction. The second column presents a scenario where both are set to lose patent 8 years after time 0. The third column presents a scenario where the comparator will lose patent 8 years after time 0 and the new drug, 14 years after time 0. The last column represents the survival profile of each treatment arm compared. For effectiveness, scenario A, assuming constant prices vs dynamic prices produces an overestimation in the cost-effectiveness ratio (ICER) of 19% for the first case, an underestimation of 13% for the second case, and an overestimation of 1% for the third case; for scenario B, assuming constant prices vs dynamic prices produces an overestimation in the ICER of 7% for the first case, an underestimation of 30% for the second case, and an overestimate of 2% for the third case; and for scenario C, assuming constant prices vs dynamic prices produces an overestimation in the ICER of 125% for the first case, an underestimation of 18% for the second case, and an overestimation of 39% for the third case.

The main results of our cost-effectiveness simulation models are presented in Figures 2 and 3. All the simulations incorporated the US generic entry pricing estimates.

The first model—the comparison between a patented originator and a generic comparator—demonstrated 3 effectiveness scenarios (mid, high, and low efficacy; Figure 2). In Figure 2, the last column presents the survival curves of treatment and control, corresponding to each drug’s survival profile, and the first 3 columns present the cumulative total cost of each therapeutic alternative (including nonpharmaceutical costs) for different patent expiration time points, ie, 2, 8, and 14 years after the baseline. The parameters for the simulation included an originator drug with an annual price of $200 000, a generic comparator with an annual price of $1000, and annual nonpharmaceutical costs of $50 000 over a 20-year time horizon discounted at a 3% rate. The model included only overall survival as the outcome of effectiveness. Then we analyzed the ICERs for each different scenario. Our model revealed that if patent expiration occurs 14 years after market entry, ignoring price dynamics after patent expiration has a very small impact on ICERs, with an overestimation (less favorable ICER) of 1%, 2%, and 5%, for the low-, mid- and high-effectiveness cases. In contrast, if patent expiration occurs only 1 year after the ICER assessment, the overestimation ranges from 33% to 40% of the ICER with constant prices.

In the second model, both drugs were under patent protection at the time of the ICER assessment. The new originator drug had an annual price of $200 000. The comparator drug had an annual price of $180 000 and annual nonpharmaceutical costs of $50 000, with the same time horizon, effectiveness, and discount rates as in the first scenario. We also analyzed 3 subscenarios: (1) the comparator drug losing its patent 2 years after time zero and the new originator drug 8 years after; (2) the patent of both drugs expiring 8 years after time zero; and (3) the patent of the comparator drug expiring 8 years after time zero, while the patent for the new originator drug expired 14 years after time zero. We found that ignoring postpatent pricing dynamics importantly affected the estimated ICERs, overestimating it by 19% to 125%, when the comparator’s patent expired before the new originator drug.

## Discussion

During the study period, drug prices decreased substantially after patent expiration across all 8 countries, from 30% to 80% over the 8 years after expiration. The simulation model indicated that not accounting for the patent expiration of originator and/or comparator drugs in cost-effectiveness analyses of new drugs produced inaccuracies in the estimation of ICERs. The findings of this evaluation can be summarized as 3 main points. First, if the new originator drug under evaluation has a high effectiveness profile and prolonged therapy administration (ie, a drug for a chronic illness administered until patient’s death or progression), ignoring postpatent price dynamics produces a less favorable than true ICER in cases where the comparator is a generic drug. Second, if the comparator drug is still under patent protection at the time of evaluation, but the patent will expire before the patent of the new originator drug, ignoring pricing dynamics will sizably overestimate the cost-effectiveness of the originator, meaning that the apparent CEA ratio is more favorable than reality. Third, the closer the time duration between CEA and patent expiration, the greater the bias in the ICER.

More specifically, in the scenario where the comparator was a generic, ICERs were underestimated. However, in the most common case—generic entry happens 14 years into the cost-effectiveness time horizon—the bias was from 1% to 5% greater than the true ICER. In scenario 2—the comparator was also under patent protection—the results varied markedly depending on the timing of the comparator patent expiration, with more favorable ICERs occurring when patent expiration was closer to time zero.

Previous studies^20^ have assessed the association of patent expiration with drug prices but they focused on smaller sample sizes, contained nonhomogeneous or historical control groups, did not account for sales data, or focused on data prior to 2009. In contrast, we applied a difference-in-differences approach with staggered patent expiration timing, used sales-unit-package-dosage−weighted prices, considered all originator drugs for each included country, and focused on price developments during the most recent decade. Additionally, several previous works^12,15^ studied case scenarios related to generic drug pricing and their effects on cost-effectiveness assessments. All of those cases studied revealed some bias when not accounting for generic pricing, despite including conservative assumptions regarding future drug prices.^12,15^

Current guidelines for health technology assessment do not typically and explicitly recommend the use of prices post−generic entry for base-case scenarios; the only exceptions are from New Zealand, Norway, the US International Society for Pharmacoeconomics and Outcomes Research, and the Second Panel on Cost Effectiveness in Health and Medicine.^15^ Our study provides relative pricing estimates that can be incorporated into all types of cost-effectiveness analyses involving drug vs drug or drug vs nondrug evaluations, either in sensitivity analyses or in base-case scenarios.^21^ Additionally, our estimates can be applied to models where both the originator and comparator are under patent protection at the time of the cost-effectiveness analysis, but the patent of the comparator drug will expire earlier than that of the new originator drug. A price decline of the comparator drug with a constant price of the new originator drug produces more favorable estimates for the cost-effectiveness of the new originator drug. When the new originator drug under evaluation has a high effectiveness profile and prolonged therapy administration, ignoring postpatent price dynamics underestimates the true ICER in cases where the comparator was a generic drug. Lastly, the closer the timeline between the CEA’s time zero and patent expiration, the greater the magnitude of the bias in the estimated ICERs in either direction.

### Limitations

Our study had limitations. First, our dataset only contained list prices for several countries. Many countries have initiated the incorporation of (confidential) rebates, for which we could not account. If such rebates increased after patent loss, our estimates are biased upwards. However, our sensitivity analyses, which included time-varying controls for volume (under the assumption that they are correlated with net prices and patent status), revealed relatively small discrepancies, indicating the robustness of the results. Second, the comparability of prices across countries is compromised due to the difference in data sources extracted by IQVIA, which include differences in data collection procedures, currency conversion, and differing national regulations on reporting. However, these differences do not bias our estimates due to each model being fitted independently for each country; however, they do hinder their cross-comparability and hence caution is required for cross-country comparisons. Third, our analysis isolated the association between patent expiration and drug prices from other factors that influence prices at the drug level, or across time, ie, before patent expiration national price negotiations or overall budget cuts. Future work should explore the drug price dynamics of these events and their incorporation into cost-effectiveness assessments.

## Conclusions

The findings of this economic evaluation indicate that drug prices declined substantially after patent expiration across these high-income countries, from 30% to 80% during the 8 years after patent expiration. The simulation model indicated that not accounting for patent expiration of comparator drugs in cost-effectiveness analyses of new drugs results in inaccuracies in the estimation of incremental cost-effectiveness ratios. These pricing dynamic estimates can be applied in base-case analyses of cost-effectiveness models for new originator drugs in Australia, Canada, France, Germany, Japan, Switzerland, UK, and US.

## Notes

Supplement 1. eAppendix. Cost-effectiveness model summary eFigure 1. Event study estimates of patent loss and prices, Callaway-Sant’Anna eTable 1. Distribution of the drugs in the study cohort classified in therapeutic areas by countries (ATC-1st-level) eTable 2. Absolute mean and median drug price levels by country, year, and patent status in US dollars (2020) eTable 3. Time distribution of observations with and without patent loss by country eFigure 2. Dynamic difference in differences estimates for the US, standard specification and time-varying volume control eFigure 3. Dynamic difference in differences estimates for Australia, standard specification and time-varying volume control eFigure 4. Dynamic difference in differences estimates for Canada, standard specification and time-varying volume control eFigure 5. Dynamic difference in differences estimates for France, standard specification and time-varying volume control eFigure 6. Dynamic difference in differences estimates for Germany, standard specification and time-varying volume control eFigure 7. Dynamic difference in differences estimates for Japan, standard specification and time-varying volume control eFigure 8. Dynamic difference in differences estimates for Switzerland, standard specification and time-varying volume control eFigure 9. Dynamic difference in differences estimates for the UK, standard specification and time-varying volume control

Supplement 2. Data Sharing Statement
