# Supplementary material for: Drug Prices After Patent Expirations in High-Income Countries and Implications for Cost-Effectiveness Analyses
Source: JAMA Health Forum. 2024 Aug 16;5(8):e242530. doi: 10.1001/jamahealthforum.2024.2530 (PMC11329876; doi:10.1001/jamahealthforum.2024.2530)
Supplement: Supplement 1. — eAppendix. Cost-effectiveness model summary eFigure 1. Event study estimates of patent loss and prices, Callaway-Sant’Anna eTable 1. Distribution of the drugs in the study cohort classified in therapeutic areas by countries (ATC-1st-level) eTable 2. Absolute mean and median drug price levels by country, year, and patent status in US dollars (2020) eTable 3. Time distribution of observations with and without patent loss by country eFigure 2. Dynamic difference in differences estimates for the US, standard specification and time-varying volume control eFigure 3. Dynamic difference in differences estimates for Australia, standard specification and time-varying volume control eFigure 4. Dynamic difference in differences estimates for Canada, standard specification and time-varying volume control eFigure 5. Dynamic difference in differences estimates for France, standard specification and time-varying volume control eFigure 6. Dynamic difference in differences estimates for Germany, standard specification and time-varying volume control eFigure 7. Dynamic difference in differences estimates for Japan, standard specification and time-varying volume control eFigure 8. Dynamic difference in differences estimates for Switzerland, standard specification and time-varying volume control eFigure 9. Dynamic difference in differences estimates for the UK, standard specification and time-varying volume control [file jamahealthforum-e242530-s001.pdf]

Supplemental Online Content

Serra-Burriel M, Martin-Bassols N, Perényi G, Vokinger KN. Patent expirations and drug price changes in high-income countries. *JAMA Health Forum.* 2024;5(8):e242530. doi:10.1001/jamahealthforum.2024.2530

eAppendix. Cost-effectiveness model summary

eFigure 1. Event study estimates of patent loss and prices, Callaway-Sant'Anna

eTable 1. Distribution of the drugs in the study cohort classified in therapeutic areas by countries (ATC-1st-level)

eTable 2. Absolute mean and median drug price levels by country, year, and patent status in US dollars (2020)

eTable 3. Time distribution of observations with and without patent loss by country

eFigure 2. Dynamic difference in differences estimates for the US, standard specification and time-varying volume control

eFigure 3. Dynamic difference in differences estimates for Australia, standard specification and time-varying volume control

eFigure 4. Dynamic difference in differences estimates for Canada, standard specification and time-varying volume control

eFigure 5. Dynamic difference in differences estimates for France, standard specification and time-varying volume control

eFigure 6. Dynamic difference in differences estimates for Germany, standard specification and time-varying volume control

eFigure 7. Dynamic difference in differences estimates for Japan, standard specification and time-varying volume control

eFigure 8. Dynamic difference in differences estimates for Switzerland, standard specification and time-varying volume control

eFigure 9. Dynamic difference in differences estimates for the UK, standard specification and time-varying volume control

This supplemental material has been provided by the authors to give readers additional information about their work.

eAppendix. Cost-effectiveness model summary

Assume that the discount rates for both costs and effectiveness are the same (r_e=r_c) and further assume that the non-pharmaceutical cost of the treatment and the control group are equal (c_{bt}=c_{at}).

$$\text{ICER} = \sum_{t=1}^{T} \frac{\frac{\Delta C_t}{(1+r_c)^{(t-1)}}}{\frac{\Delta E_t}{(1+r_e)^{(t-1)}}} = \sum_{t=1}^{T} \frac{\frac{(p_{bt}+c_{bt})*s_{bt}-(p_{at}+c_{at})*s_{at}}{(1+r_c)^{(t-1)}}}{\frac{s_{bt}-s_{at}}{(1+r_e)^{(t-1)}}} = \sum_{t=1}^{T} \frac{p_{bt}*s_{bt}-p_{at}*s_{at}}{s_{bt}-s_{at}} + c_{bt}$$

In the last step the two assumptions were used namely that $\frac{(1+r_c)^{(t-1)}}{(1+r_e)^{(t-1)}} = 1$ and that

$$\frac{p_{bt}*s_{bt}+c_{bt}*s_{bt}-p_{at}*s_{at}-c_{at}*s_{at}}{s_{bt}-s_{at}} = \frac{p_{bt}*s_{bt}-p_{at}*s_{at}}{s_{bt}-s_{at}} + \frac{c_{bt}*(s_{bt}-s_{at})}{s_{bt}-s_{at}} = \frac{p_{bt}*s_{bt}-p_{at}*s_{at}}{s_{bt}-s_{at}} + c_{bt}$$

Further assuming that c_{bt} and p_{at} are constant over time (c_b, p_a) we can simplify to:

$$\sum_{t=1}^{T} \frac{p_{bt}*s_{bt}-p_a*s_{at}}{s_{bt}-s_{at}} + c_b = \sum_{t=1}^{T} \frac{p_{bt}*s_{bt}-p_a*s_{at}}{s_{bt}-s_{at}} + c_b + \frac{p_a*s_{bt}}{s_{bt}-s_{at}} - \frac{p_a*s_{bt}}{s_{bt}-s_{at}}$$

$$= \sum_{t=1}^{T} \frac{p_{bt}*s_{bt}}{s_{bt}-s_{at}} + \frac{p_a*s_{bt}-p_a*s_{at}}{s_{bt}-s_{at}} - \frac{p_a*s_{bt}}{s_{bt}-s_{at}} + c_b$$

$$= \left[\sum_{t=1}^{T} \frac{p_{bt}*s_{bt}}{s_{bt}-s_{at}}\right] - p_a * \left[\sum_{t=1}^{T} \frac{s_{bt}}{s_{bt}-s_{at}}\right] - T*(p_a+c_b)$$

Using in the last step that we can some over p_a and c_b as they are constant in time.

Finally, by recentering the observations we can set p_a and c_b to 0, meaning that the control group has assumed cost zero, and then derive the final expression for ICER:

$$\text{ICER} = \left[\sum_{t=1}^{T} \frac{p_{bt}*s_{bt}}{s_{bt}-s_{at}}\right]$$

eFigure 1. Event study estimates of patent loss and prices, Callaway-Sant'Anna

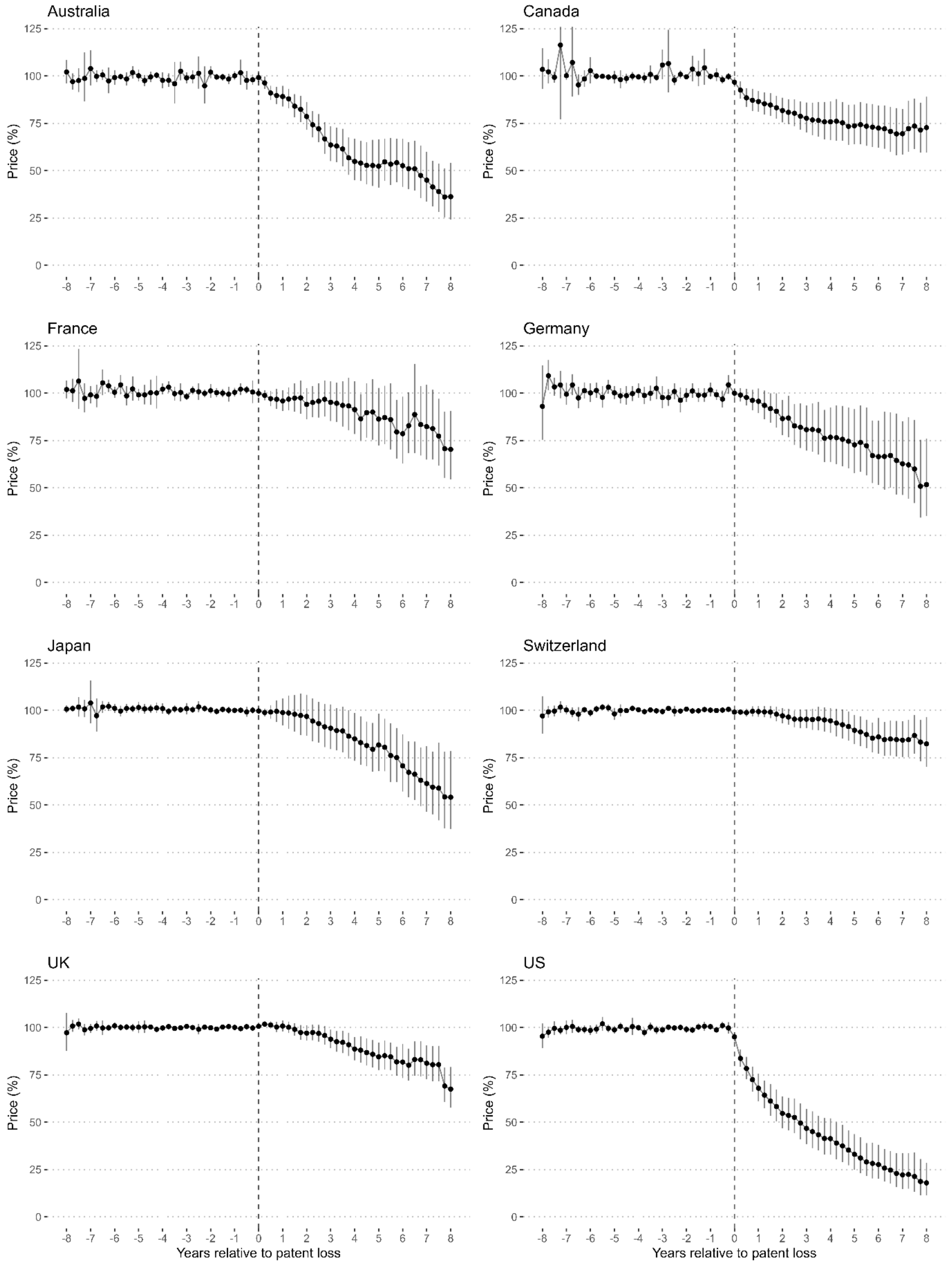

Notes: The time unit of estimation is at the molecule-quarter level. Standard errors are clustered at the molecule level, lines present 95% confidence intervals.

eTable 1. Distribution of the drugs in the study cohort classified in therapeutic areas by countries (ATC-1st-level)

ATC group code	Australia	Canada	France	Germany	Japan	Switzerland	UK	US
Alimentary tract and metabolism	46	37	26	35	52	30	35	56
Blood and blood forming organs	18	21	18	26	15	19	25	39
Cardiovascular system	21	35	25	35	31	38	33	40
Dermatologicals	9	10	4	8	9	5	7	11
Genito urinary system and sex hormones	12	8	11	9	7	10	10	15
Systemic hormonal preparations, excl. sex hormones and insulins	4	7	6	9	5	4	5	8
Anti-infective for systemic use	57	68	69	70	62	61	65	83
Antineoplastic and immunomodulating agents	88	94	96	106	97	86	97	118
Musculo-skeletal system	15	10	11	10	11	14	15	18
Nervous system	36	36	25	32	31	32	36	63
Respiratory system	19	18	16	20	17	14	18	21
Sensory organs	8	15	7	9	12	7	8	12
Various	5	2	6	9	6	3	5	13

Notes: ATC: anatomical therapeutic categories

eTable 2. Absolute mean and median drug price levels by country, year, and patent status in US dollars (2020)

		Australia		Canada		France		Germany		Japan		Switzerland		UK		US	
Year	Patent	Mean	Median	Mean	Median	Mean	Median	Mean	Median	Mean	Median	Mean	Median	Mean	Median	Mean	Median
2011	Protected	1254.7	680.3	2446.6	1010.4	2302.3	61.4	2399.3	536.6	1293.4	941.1	1969.4	15.8	1844.5	873.8	2556.3	589.6
2012	Protected	1256.9	603.2	2721.8	1459.5	1478.7	66.9	2930.4	1031.4	1482.6	865.1	2182.9	132.5	2410.3	867.7	3185.4	906.3
2013	Protected	1300.9	568.2	2634.7	1331.3	1342	150.5	3180.3	1265.9	1116	662.4	2161.5	150	2299	839.3	4031.2	975.5
2014	Protected	1535.1	620.4	2898.3	1361.6	1310.8	315.5	3014	1217.9	1068	707.6	2444.8	158.4	2621.7	904.4	4724	1526.5
2015	Protected	1319	380.8	2717	1186.8	1330	347.3	2828.9	1300.4	1235	644	2576.5	692.5	2533.6	1177.1	4745.5	1253.3
2016	Protected	1466.3	391.8	2576.6	1092.7	1359	260.6	2937.4	1290.8	1369.9	714.5	2375.1	1039.6	2208.6	1075.3	4808.1	1439.5
2017	Protected	1697.7	534.1	2720.6	1058.3	1385.8	587.1	2939.9	1428.8	1149.4	725.9	2634.1	1268.2	2243.8	1021.7	5089.4	1737.9
2018	Protected	1634.7	475.4	2709	1073	2162.7	876.9	2973.9	1485.6	1273.9	806.2	2464.7	1180.3	2408.6	1060.3	5096.8	2040.7
2019	Protected	1536	440.1	2417.6	939.4	2137	907.8	2718.1	1324	1413.5	843.6	2219.9	1045.9	2352.3	1002	5395.2	2433.1
2020	Protected	1666.3	456.9	2588.2	1001.1	2227.4	899.1	2936.4	1413.3	1418.4	871.2	2435.3	1122.2	2434.1	1044.5	5586.8	2541
2011	Unprotected	536.4	110.7	738	173.3	599.3	60.1	845.5	135.5	576.3	249.7	963.2	192.4	587.4	128.5	1133.4	354.2
2012	Unprotected	585.9	117.3	840.9	184.1	586.6	57.5	851.4	132.8	552.1	242	996.1	184.1	605.4	181.6	1267.1	341
2013	Unprotected	525.3	88.1	828.1	174.2	570.6	60.8	931.9	159.5	440.9	193.2	970.3	181.9	691.4	189.9	1361.7	353.6
2014	Unprotected	454.2	69.8	787.9	172.9	610.8	61.2	972.8	140.1	427	167.4	999.8	188.1	743.8	189.2	1392.7	382.8
2015	Unprotected	339.1	51.8	676.3	148	490.3	50.1	984.6	121.1	412.9	147.5	1078.2	186.2	726.3	181.1	1734.8	463.3
2016	Unprotected	334.9	49.3	654.9	132.6	513.6	57.3	881.9	120.9	489	148.4	1038.3	204.8	726	149.8	1902	530.3
2017	Unprotected	354.1	48.9	663	129	597.9	62.6	861	132.5	506.4	137.5	1032.9	207.1	730.6	122	2097.2	519.7
2018	Unprotected	339.7	45.1	708.4	128.5	625.5	59.5	923.1	125.2	507.7	132.9	939.4	176.7	786.8	141.1	2103	431.7
2019	Unprotected	338.8	39.9	694.3	118.1	595	56.9	883.7	115.8	493.4	123.7	883.2	161.3	803.8	127.7	1997.6	397.6
2020	Unprotected	315.3	35.2	690.5	120.1	571.7	57.4	833.4	116.2	492.2	119.6	922.8	162	817.7	121.4	1776.9	387

Notes: Unprotected drugs class includes substances that lost patent protection in a given year alongside those that did before that given year

eTable 3. Time distribution of observations with and without patent loss by country

	**Australia**	**Canada**	**France**	**Germany**	**Japan**	**Switzer-land**	**UK**	**US**
Time	Treated	Treated	Treated	Treated	Treated	Treated	Treated	Treated
-32	17	13	15	26	13	17	17	24
-31	18	16	18	29	16	20	20	27
-30	19	18	20	31	18	25	23	34
-29	21	24	24	35	21	30	28	40
-28	24	31	28	39	27	33	30	43
-27	33	32	29	41	30	37	33	49
-26	38	40	36	48	33	43	38	52
-25	47	44	41	52	38	46	41	54
-24	52	45	45	56	40	48	43	59
-23	55	48	47	59	43	50	47	64
-22	57	50	49	61	47	52	49	74
-21	63	54	52	65	50	55	54	81
-20	66	59	56	66	59	56	58	84
-19	73	68	62	73	62	62	66	100
-18	83	73	66	79	69	64	70	105
-17	88	80	73	90	72	68	76	116
-16	90	84	78	95	79	73	82	122
-15	94	89	84	101	82	83	85	130
-14	100	100	91	108	87	91	94	136
-13	111	104	94	110	94	93	98	152
-12	117	106	95	112	98	95	99	167
-11	123	110	102	120	105	103	104	177
-10	128	120	113	127	116	114	113	183
-9	134	126	120	135	121	117	121	193
-8	144	129	123	139	125	122	123	196
-7	150	138	125	142	132	123	127	200
-6	155	144	131	145	138	126	132	208
-5	159	150	138	149	146	131	138	224
-4	168	160	147	159	155	140	144	235
-3	174	171	149	163	162	144	149	246
-2	184	190	160	175	169	154	158	256
-1	184	190	160	175	169	154	158	256
0	191	204	173	187	183	169	172	272
1	188	200	168	182	185	166	171	265
2	185	195	164	176	180	160	169	261
3	177	186	161	174	178	153	159	257
4	171	186	161	170	178	149	156	251
5	168	182	149	159	175	140	148	247
6	163	182	149	159	174	138	148	244
7	161	175	142	152	172	131	145	242
8	160	173	140	152	169	134	145	240
9	161	172	137	149	165	132	143	241
10	160	170	133	148	162	129	142	232
11	159	164	129	141	161	127	137	230
12	155	160	128	139	156	126	135	226
13	145	155	128	137	154	125	134	217
14	142	146	125	128	150	115	130	215
15	133	145	119	127	142	113	126	213
16	127	142	120	125	141	111	124	208
17	122	140	119	124	142	110	121	205
18	121	140	119	122	139	105	120	193
19	114	140	115	121	137	107	117	187
20	113	138	116	118	124	103	111	181
21	107	130	109	111	121	98	105	162

22	99	127	109	107	115	96	99	155
23	96	120	99	101	111	91	94	146
24	95	112	94	95	106	88	87	143
25	90	107	89	90	102	83	86	135
26	85	98	78	80	96	72	75	128
27	72	95	77	78	90	73	75	116
28	68	91	77	76	87	69	73	101
29	63	84	66	67	80	64	67	92
30	59	78	59	61	66	55	60	87
31	52	72	52	52	61	51	53	79
32	44	70	47	47	59	49	50	76
Control†	146	149	128	174	156	154	179	222
Ratio	1.31	1.37	1.35	1.07	1.19	1.1	0.96	1.23

Notes: † never treated, i.e., never experienced patent loss in the observation window.

eFigure 2. Dynamic difference in differences estimates for the US, standard specification and time-varying volume control

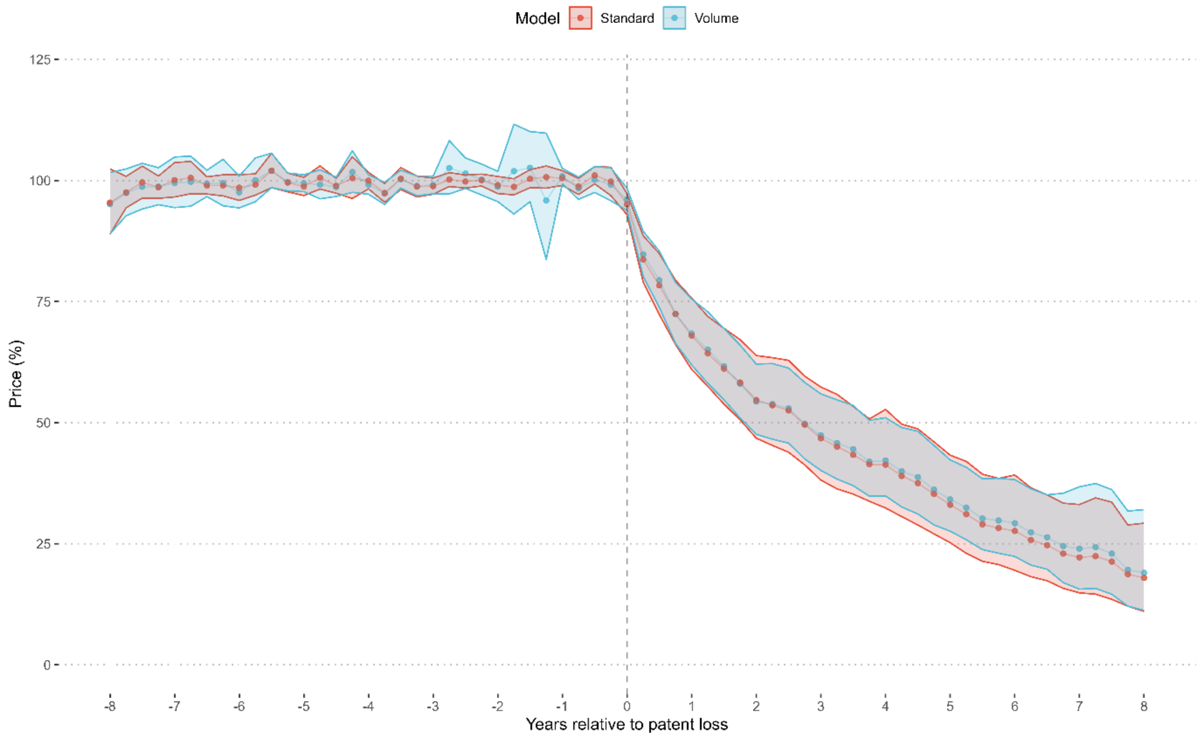

Notes: dots present point estimates and their associated ribbon the 95%CI.

eFigure 3. Dynamic difference in differences estimates for Australia, standard specification and time-varying volume control

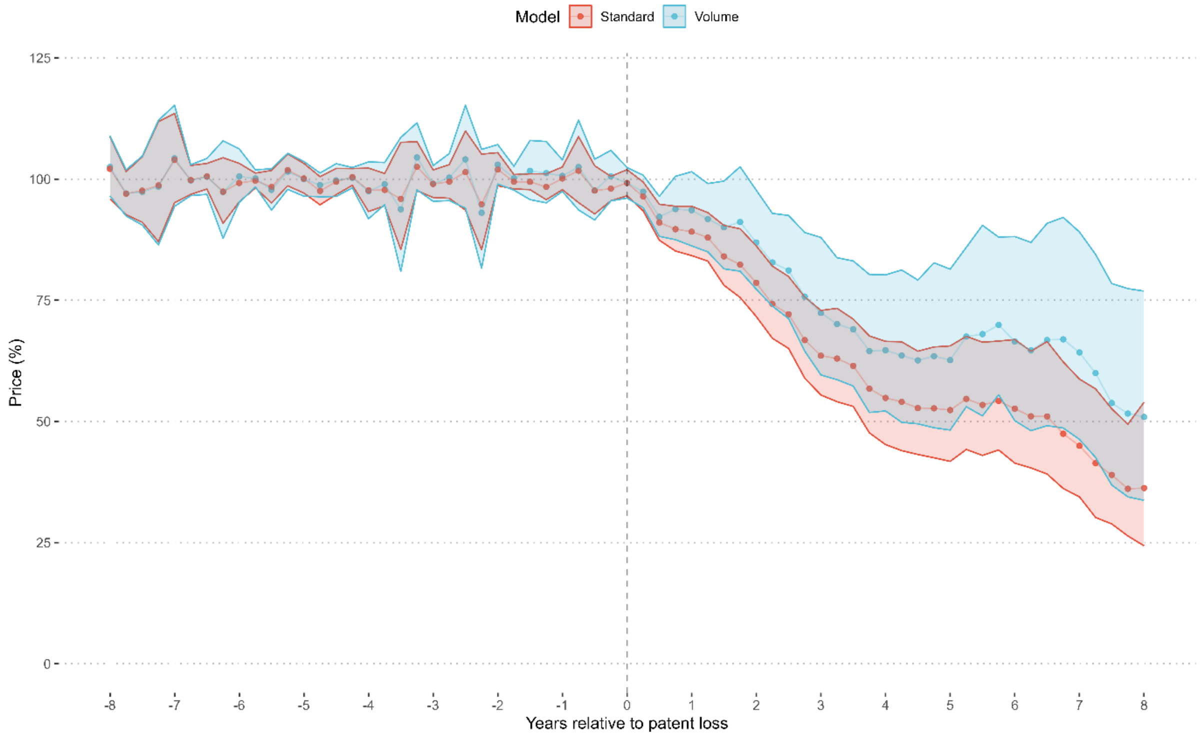

Notes: dots present point estimates and their associated ribbon the 95%CI.

eFigure 4. Dynamic difference in differences estimates for Canada, standard specification and time-varying volume control

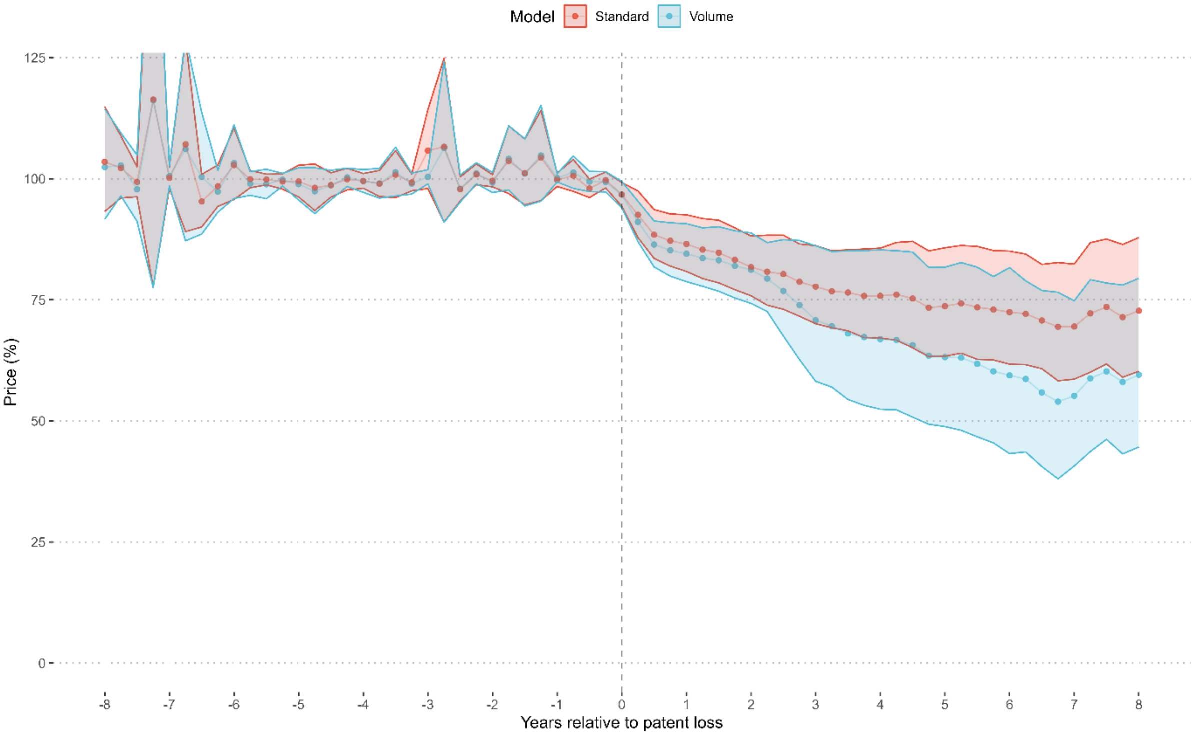

Notes: dots present point estimates and their associated ribbon the 95%CI.

eFigure 5. Dynamic difference in differences estimates for France, standard specification and time-varying volume control

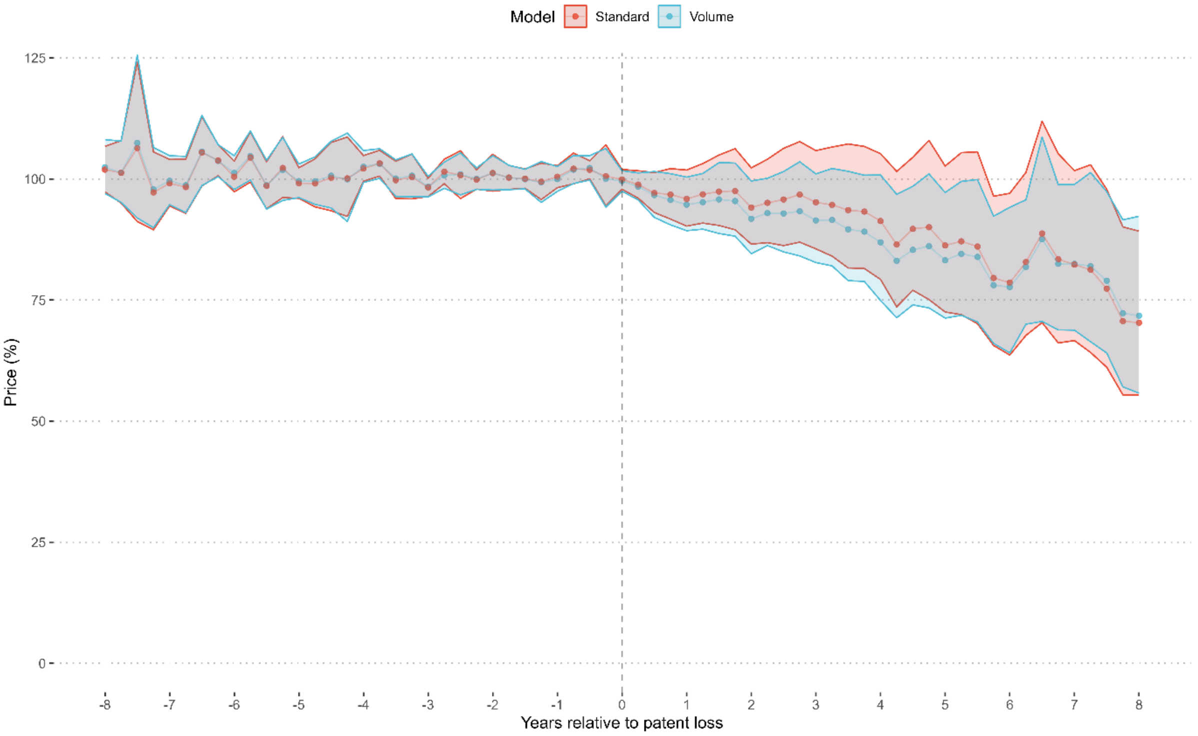

Notes: dots present point estimates and their associated ribbon the 95%CI.

eFigure 6. Dynamic difference in differences estimates for Germany, standard specification and time-varying volume control

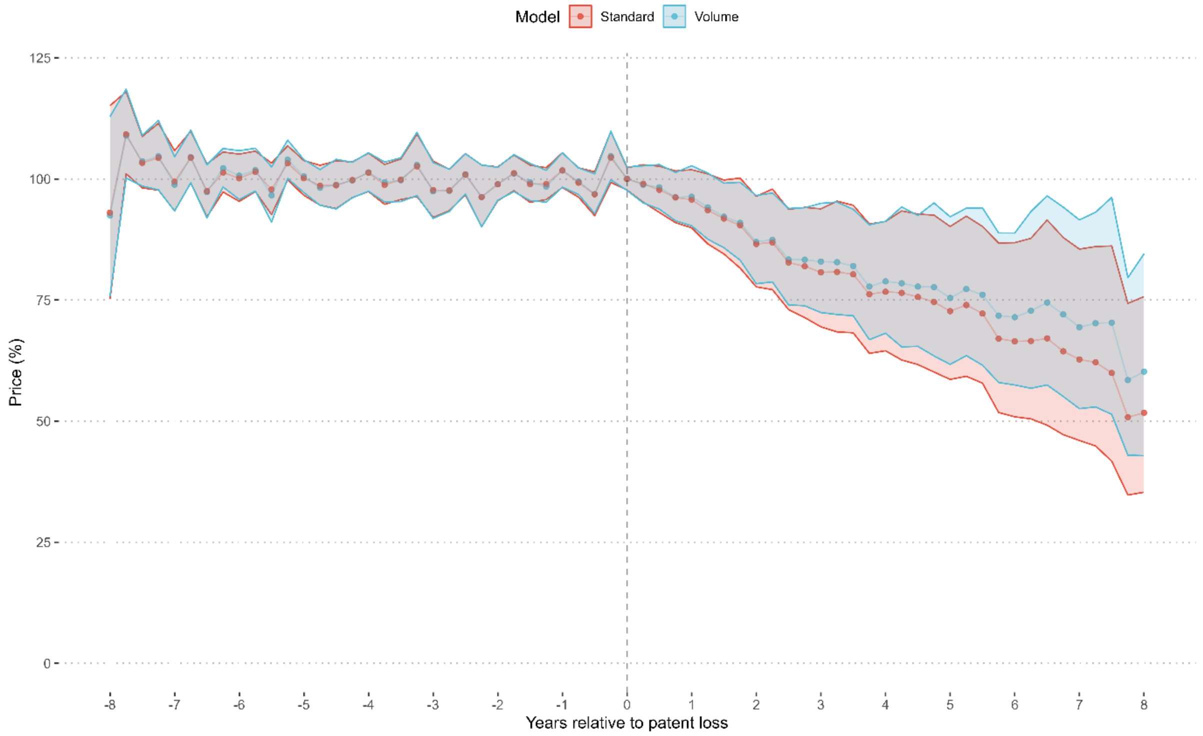

Notes: dots present point estimates and their associated ribbon the 95%CI.

eFigure 7. Dynamic difference in differences estimates for Japan, standard specification and time-varying volume control

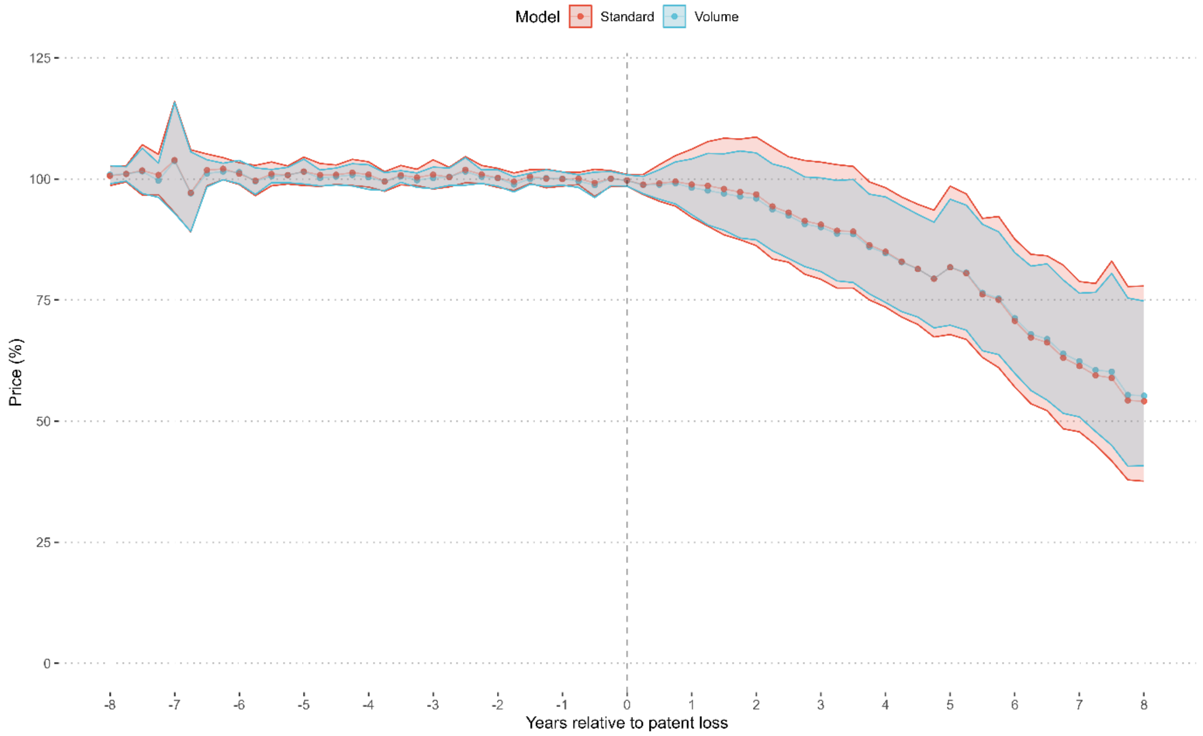

Notes: dots present point estimates and their associated ribbon the 95%CI.

eFigure 8. Dynamic difference in differences estimates for Switzerland, standard specification and time-varying volume control

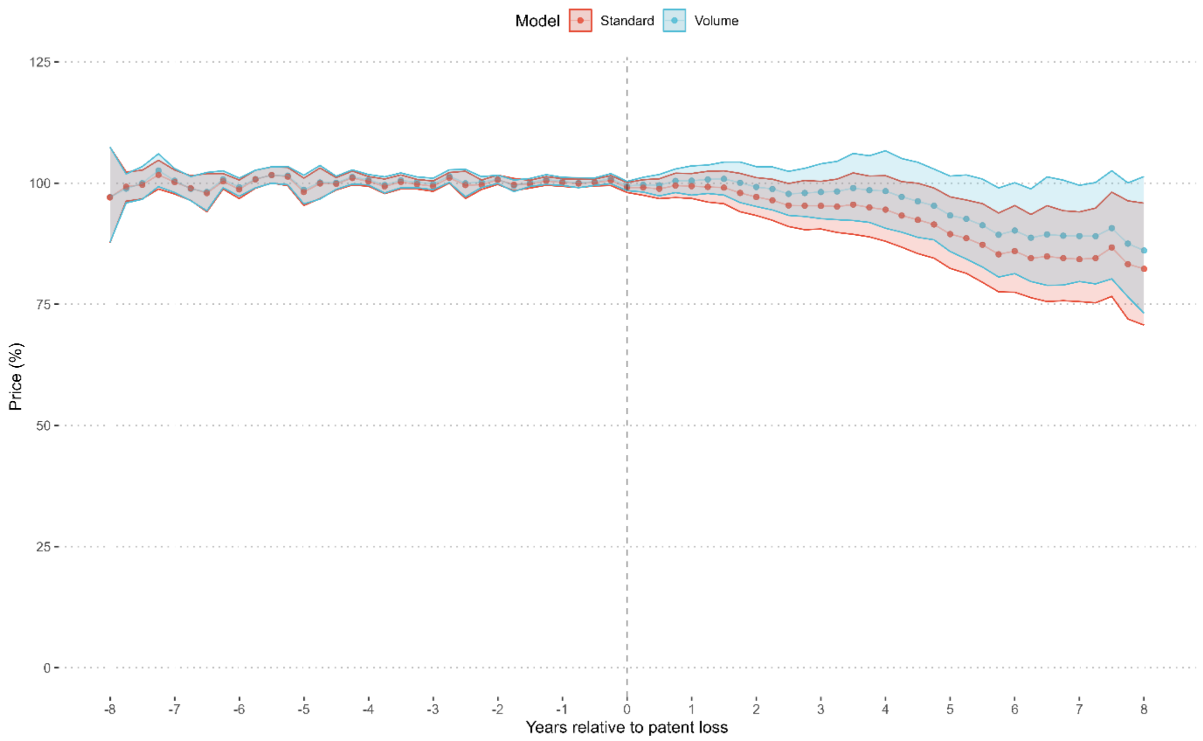

Notes: dots present point estimates and their associated ribbon the 95%CI.

eFigure 9. Dynamic difference in differences estimates for the UK, standard specification and time-varying volume control

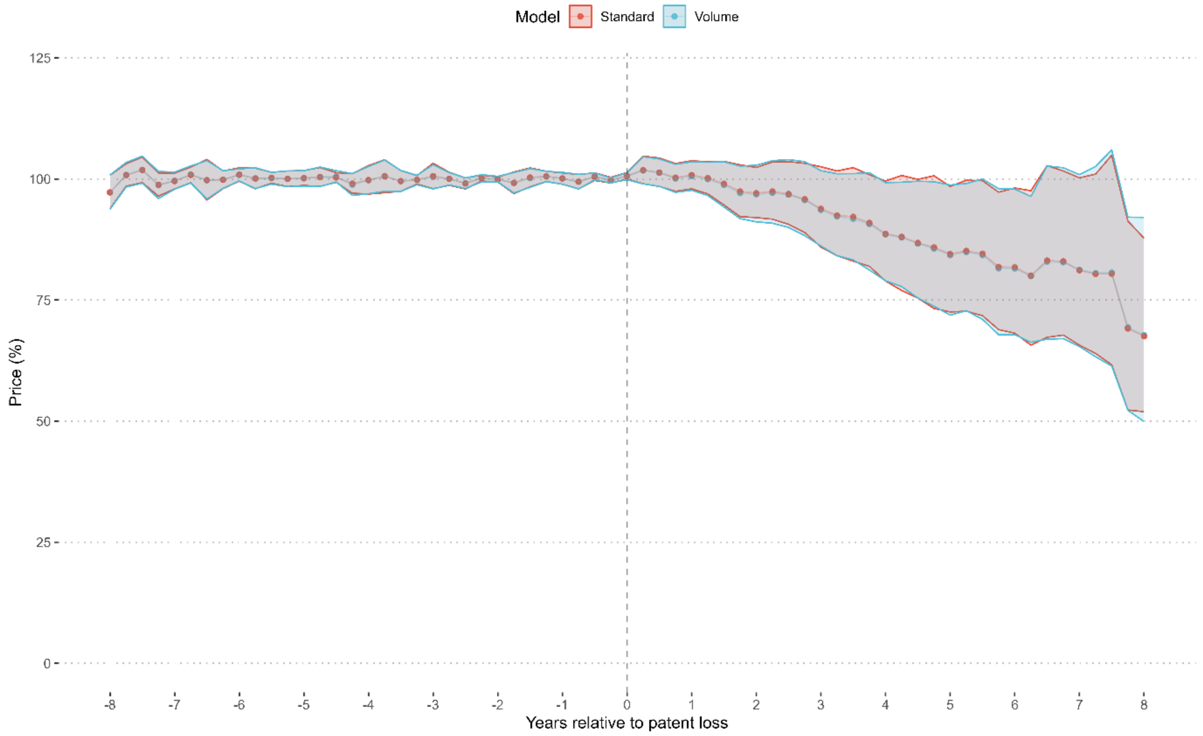

Notes: dots present point estimates and their associated ribbon the 95%CI.
