# Supplementary material for: Drug Prices After Patent Expirations in High-Income Countries and Implications for Cost-Effectiveness Analyses
Source: JAMA Health Forum. 2024 Aug 16;5(8):e242530. doi: 10.1001/jamahealthforum.2024.2530 (PMC11329876; doi:10.1001/jamahealthforum.2024.2530)
Supplement: Supplement 2. — Data Sharing Statement [file jamahealthforum-e242530-s002.pdf]

Data Sharing Statement

Serra-Burriel. Drug Prices After Patent Expirations in High-Income Countries and Implications for Cost-Effectiveness Analyses. *JAMA Health Forum*. Published August 16, 2024. doi:10.1001/jamahealthforum.2024.2530

Data
Data available: No
